# Bdh1 overexpression ameliorates hepatic injury by activation of Nrf2 in a MAFLD mouse model

**DOI:** 10.1038/s41420-022-00840-w

**Published:** 2022-02-03

**Authors:** Bu-tuo Xu, Fang-yuan Teng, Qi Wu, Sheng-rong Wan, Xin-yue Li, Xiao-zhen Tan, Yong Xu, Zong-zhe Jiang

**Affiliations:** 1 Department of Endocrinology and Metabolism, The Affiliated Hospital of Southwest Medical University, Luzhou, Sichuan 646000 China; 2 Metabolic Vascular Disease Key Laboratory of Sichuan Province, Luzhou, Sichuan 646000 China; 3 Sichuan Clinical Research Center for Nephropathy, Luzhou, Sichuan 646000 China; 4 Department of Pathology, Academician (Expert) Workstation of Sichuan Province, the Affiliated Hospital of Southwest Medical University, Luzhou, Sichuan 646000 China

**Keywords:** Non-alcoholic fatty liver disease, Apoptosis

## Abstract

In 2020, a group of experts officially suggested metabolic dysfunction associated with fatty liver disease “MAFLD” as a more appropriate overarching term than NAFLD, indicating the key role of metabolism in fatty liver disease. Bdh1, as the rate-limiting enzyme of ketone metabolism, acts as an important metabolic regulator in liver. However, the role of Bdh1 in MAFLD is unclear. In this study, we used the transgenic db/db mice as a MAFLD mouse model and observed the downregulated expression of Bdh1 in fatty liver. In addition, expression of Bdh1 was also reduced by palmitic acid (PA) treatment in LO2 cells. Bdh1 knockdown led to ROS overproduction and ROS-induced inflammation and apoptosis in LO2 cells, while Bdh1 overexpression protected LO2 cells from lipotoxicity by inhibiting ROS overproduction. Mechanistically, Bdh1-mediated βOHB metabolism inhibits ROS overproduction by activation of Nrf2 through enhancement of metabolic flux composed of βOHB-AcAc-succinate-fumarate. Notably, adeno-associated virus (AAV)-mediated Bdh1 overexpression successfully reversed the hepatic function indexes, fibrosis, inflammation, and apoptosis in fatty livers from db/db mice. In conclusion, our study revealed a Bdh1-mediated molecular mechanism in pathogenesis of metabolic dysfunction related liver disease and identified Bdh1 as a novel potential therapeutic target for MAFLD.

## Introduction

In 2018, the world population with NAFLD has reached 25% [1]. Approximately 10–20% of NAFLD cases progress to NASH, which can ultimately progress to cirrhosis, hepatocellular carcinoma (HCC), and death [2]. In recent years, an increasing number of evidence shows that the name “NAFLD” can not reflect the characteristics of widely combined metabolic disorders in patients [3]. In addition, the development of NASH is typically accompanied by factors related to metabolic syndrome, especially the presence of Obesity and T2DM [4–6]. Therefore, in 2020, a group of experts officially renamed NAFLD metabolic associated fatty liver disease (MAFLD) to emphasize the key role of metabolic disorders [7]. Unfortunately, to date, there are no available pharmacotherapies for treating NASH [8]. Thus, preventing the development of severe liver disease by controlling the progression of MAFLD is urgently needed.

As a complex and multifactorial disease, the exact pathogenesis of MAFLD is still unclear [9]. At present, the newly proposed theory called the “multiple hits” hypothesis, corresponding to the previous “two hit” hypothesis, involves many factors that may act in parallel but not in turn, which provides a more appropriate demonstration of the pathogenesis of MAFLD [10]. As one of the various factors that contribute to the “multiple hits”, oxidative stress is considered the dominant contributor to the disease progression in MAFLD [11]. Under normal physiological conditions, the production and elimination of reactive oxygen species (ROS) reach a dynamic balance, which would be impaired and exhibited as oxidative stress in diseases, such as MAFLD [12]. In different types of liver cells, ROS are continuously produced intracellularly as byproducts of energetic metabolism [13]. Hepatic lipid accumulation induces the overproduction of ROS by promoting ROS-generating mechanisms, including fatty acid oxidation (FAO) [14]. At high concentrations, ROS cause the accumulation of damaged oxidative modification in DNA, lipids, and proteins, leading to inflammation, fibrosis, and apoptosis in liver [15, 16]. It is well known that the transcription factor nuclear factor red 2-related factor 2 (Nrf2) can maintain intracellular redox homeostasis and reduce cell damage by regulating the expression of antioxidant proteins [17, 18]. In a high-fat-diet-induced MAFLD mouse model, it was reported that Nrf2 deficiency is associated with ROS overproduction and glutathione level decrease, which led to accelerated progression to NASH [19]. Fumarate, an intermediate product of the TCA cycle, is well known to activate Nrf2-mediated antioxidant response [20]. However, the role of metabolic regulation in the Nrf2-mediated anti-ROS pathway and the pathogenesis of MAFLD is still unclear.

Ketone body is mainly composed of β-Hydroxybutyrate (βOHB) which accounts for 80%. Except for being an alternative energy source, βOHB can also function in processes of anti-oxidative stress, anti-inflammation and anti-aging [21–23]. β-hydroxybutyrate dehydrogenase 1 (Bdh1), the rate-limiting enzyme of ketone body metabolism, can directly catalyze the metabolism of βOHB and promote the reciprocal transformation between βOHB and AcAc [23]. In TAC-induced heart failure, Bdh1 cardiac overexpression can significantly ameliorate the pathogenesis through inhibition of oxidative stress [24]. In liver, deletion of Bdh1 causes low ketone body level and fatty liver during fasting [25]. Moreover, Bdh1-mediated β-hydroxybutyrylation potentiates propagation of hepatocellular carcinoma stem cells [26]. However, the role of Bdh1-mediated ketone body metabolism in the pathogenesis of MAFLD is still unknown.

In this study, we report that Bdh1 deficiency is related to the pathogenesis of MAFLD in vivo and lipotoxicity in vitro. We also demonstrate that Bdh1 inhibits lipotoxicity-induced ROS overproduction by activation of Nrf2 through enhancement of metabolic flux composed of βOHB-AcAc-succinate-fumarate. Notably, the AAV-mediated hepatic expression of Bdh1 effectively relieved the progression of MAFLD. Taken together, our findings suggest a promising new therapy for MAFLD via targeting Bdh1-mediated antioxidant pathway.

## Results

### Bdh1 was downregulated in livers from MAFLD mouse model and palmitic acid (PA)-treated LO2 cells

**Fig. 1:**
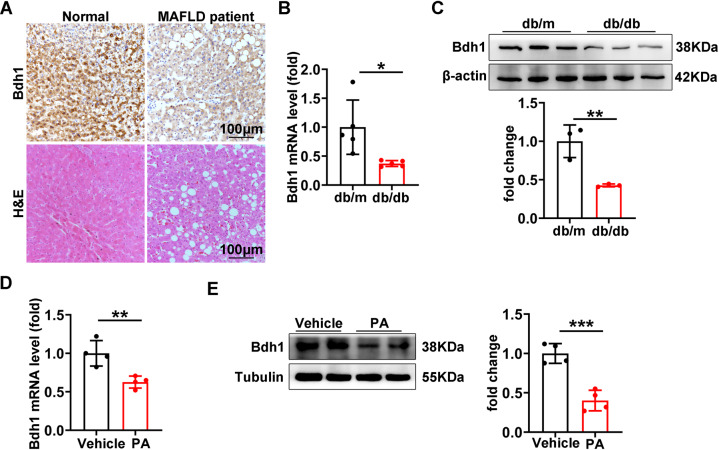
Bdh1 expression is downregulated in liver of the MAFLD mouse model. **A** Representative images showing immunohistochemical staining of Bdh1 in liver tissue from three normal donors and from three individuals with MAFLD. Bar: 100 μm. **B** qRT-PCR analysis showing the mRNA level of Bdh in livers from db/m and db/db mice. **C** Western blot showing the protein level of Bdh1 in livers from db/m and db/db mice. **D** qRT-PCR analysis showing the mRNA level of Bdh in LO2 cells with vehicle and palmitic acid treatment. **E** Western blot showing the protein level of Bdh1 in LO2 cells with vehicle and palmitic acid (150 μmol) treatment. All results are representative of three independent experiments. Values are presented as mean ± SD. *P < 0.05; **P < 0.01; ***P < 0.001.

To investigate whether Bdh1 was involved in the pathogenesis of MAFLD, we firstly detected the expression levels of Bdh1 in normal and MAFLD patients. Interestingly, expression of Bdh1 in livers from MAFLD patients was significantly reduced (Fig. 1A). we next detected the expression levels of Bdh1 in livers from db/m and db/db mice, a classic genetic model of obesity-associated fatty liver. As shown in Fig. 1B, C, either mRNA level or protein level of Bdh1 was reduced in MAFLD mouse model, suggesting that the expression change of Bdh1 was associated with progression of MAFLD. As it is known, hyperlipidemia is the most obvious characteristic of fatty liver disease [4]. In view of this, we established PA-induced lipotoxicity cell model with LO2 cells to evaluate the effect of PA on the Bdh1 expression. As expected, either mRNA level or protein level of Bdh1 was obviously reduced by PA treatment in LO2 cells (Fig. 1D, E), indicating that the hyperlipidemia-induced Bdh1 deficiency might contribute to the pathogenesis of MAFLD.

### Bdh1 deficiency promoted PA-induced inflammation and apoptosis by ROS overproduction

**Fig. 2:**
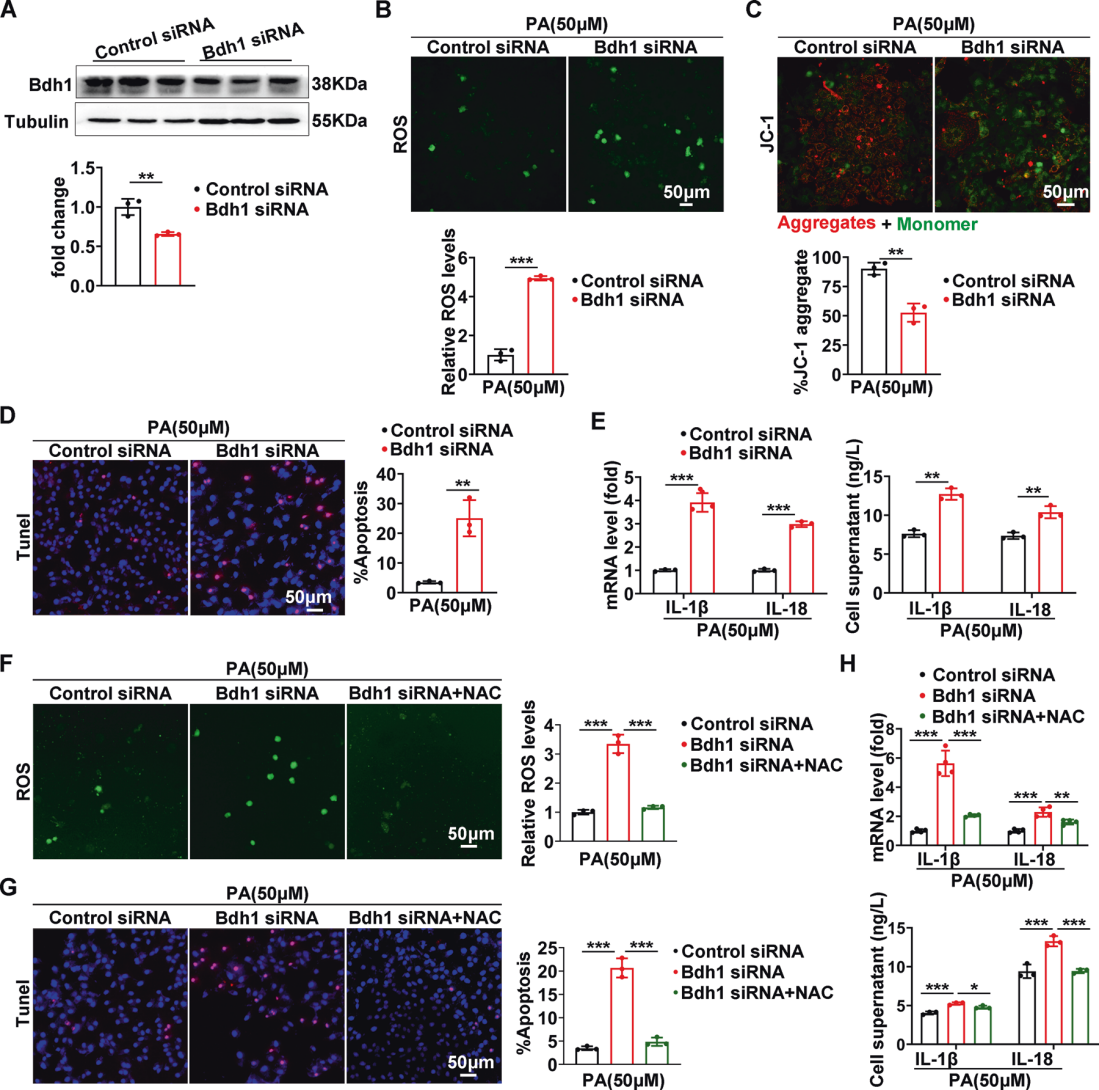
Bdh1 knockdown promoted PA-induced inflammation and apoptosis by overproduction of ROS in LO2 cells. **A** Representative western blots and quantification of Bdh1 expression in the LO2 cells transfected with control siRNA or Bdh1 siRNA. **B** DCFH-DA probe was used to detect the level of ROS in LO2 cells transfected with control siRNA or Bdh1 siRNA. All the cells were treated with 50 μmol PA for 24 h before ROS assay. **C** JC-1 staining showing the mitochondrial membrane potential in LO2 cells transfected with control siRNA or Bdh1 siRNA. **D** TUNEL assay showing the apoptosis level of LO2 cells transfected with control siRNA or Bdh1 siRNA. All the cells were treated with 50 μmol PA for 24 h before TUNEL assay. **E** mRNA levels (above) and cell culture supernatant concentrations (below) of IL-1β and IL-18 in LO2 cells transfected with NC siRNA or Bdh1 siRNA. **F** DCFH-DA probe was used to detect the level of ROS in LO2 cells transfected with control siRNA or Bdh1 siRNA and Bdh1 siRNA-transfected LO2 cells with NAC treatment. All the cells were treated with 50 μmol PA for 24 h before ROS assay. **G** TUNEL assay showing the apoptosis level of LO2 cells transfected with control siRNA or Bdh1 siRNA and Bdh1 siRNA-transfected LO2 cells with NAC treatment. All the cells were treated with 50 μmol PA for 24 h before TUNEL assay. **H** mRNA levels (above) and cell culture supernatant concentrations (below) of IL-1β and IL-18 in LO2 cells transfected with control siRNA or Bdh1 siRNA and Bdh1 siRNA-transfected LO2 cells with NAC treatment. All results are representative of three independent experiments. Bar: 50 μm. Values are presented as mean ± SD. *P < 0.05; **P < 0.01; ***P < 0.001.

To investigate whether the Bdh1 reduction contributes to PA-induced cell injury, we performed Bdh1 knockdown in LO2 cells (Fig. 2A). Given that the increased ROS plays a central and prominent role in the pathogenesis of fatty liver disease [15] and Bdh1 have been reported to inhibit oxidative stress in heart failure [24], we next detected the ROS level and observed a significant increase of ROS in LO2 cells transfected with Bdh1 siRNA (Fig. 2B). We also found that the mitochondrial membrane potential was reduced by Bdh1 knockdown with JC-1 staining (Fig. 2C), which indicates that the total ROS was mainly from mitochondrial ROS. Notably, TUNEL assay showed that the Bdh1 knockdown also remarkably increased PA-induced apoptosis (Fig. 2D). In addition, the protein level of activated proinflammatory factors, cleaved IL-1β and IL-18, were also elevated by Bdh1 knockdown, as well as the secretory IL-1β and IL-18 (Fig. 2E).

To further investigate whether the Bdh1 reduction promotes apoptosis and inflammation by overproducing ROS, we used ROS inhibitor, NAC, to treat LO2 cells which were transfected with Bdh1 siRNA. ROS assay showed the NAC totally blocked the PA-induced ROS overproduction in LO2 cells transfected with Bdh1 siRNA (Fig. 2F). Of note, NAC also reversed the Bdh1 knockdown-mediated apoptosis increase (Fig. 2G), as well as the increase of IL-1β and IL-18 (Fig. 2H). Collectively, these results suggest that Bdh1 deficiency might mediate PA-induced cell apoptosis and inflammation by loss of anti-ROS function.

### Bdh1 overexpression reversed PA-induced ROS overproduction, inflammation, and apoptosis

**Fig. 3:**
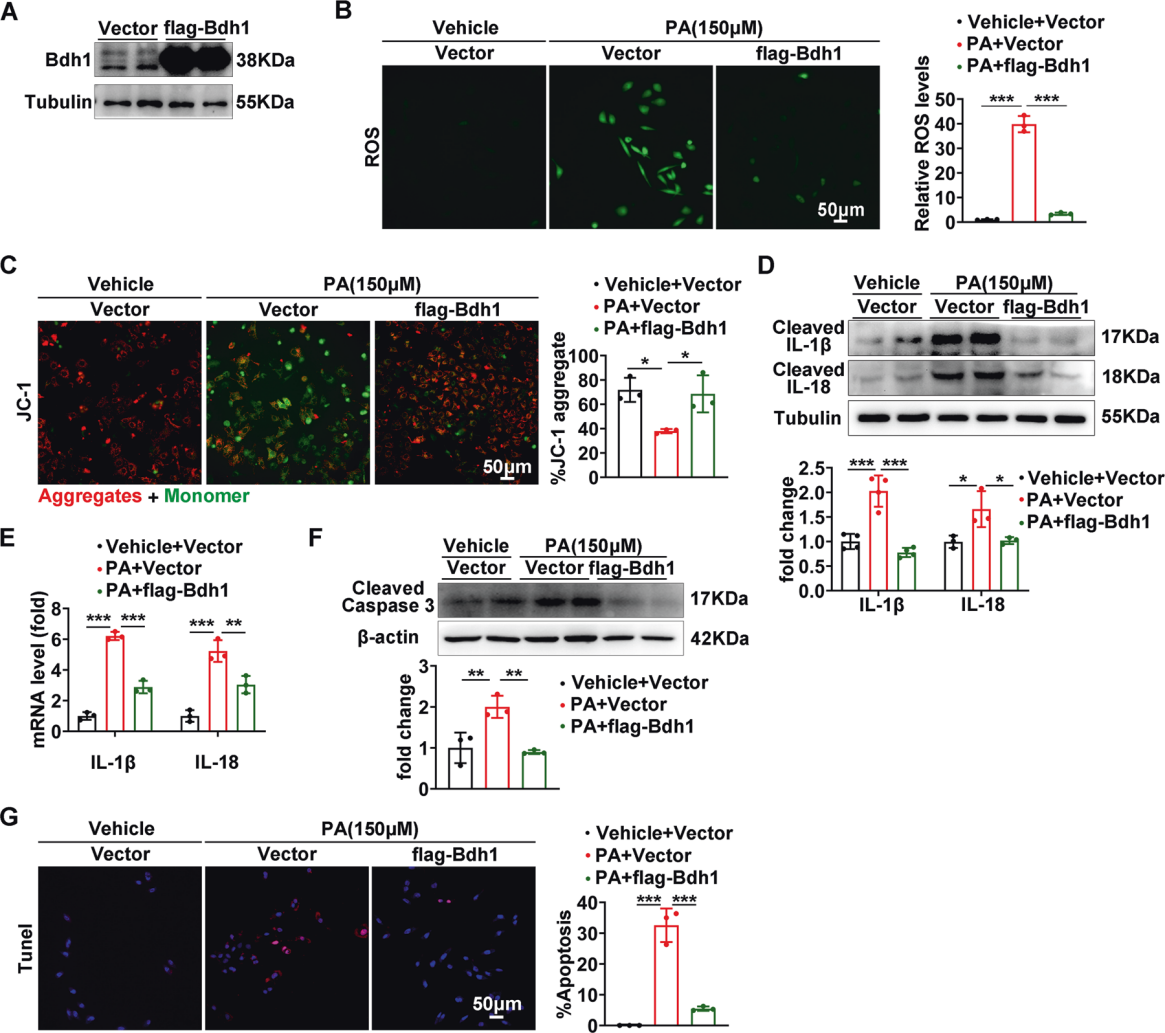
Bdh1 overexpression reversed PA-induced ROS overproduction, inflammation, and apoptosis. **A** Representative western blots showing the protein level of Bdh1 in LO2 cells transfected with vector or flag-Bdh1. **B** DCFH-DA probe was used to detect the level of ROS in LO2 cells with indicated treatment. Bar: 50 μm in upper panels and 100 μm in lower panels. Before ROS assay, LO2 cells were transfected with the plasmids overexpressing flag-Bdh1 or vector for 24 h, and then treated with vehicle or PA for 24 h. **C** JC-1 staining showing the mitochondrial membrane potential in LO2 cells with indicated treatment. **D** Representative Western blots showing the protein level of IL-1β and IL-18 in LO2 cells with indicated treatment. **E** mRNA levels of IL-1β and IL-18 in LO2 cells with indicated treatment. **F** Representative western blots showing the protein level of cleaved caspase 3 in LO2 cells with indicated treatment. **G** TUNEL assay showing the apoptosis level of LO2 cells with indicated treatment. Before TUNEL assay, LO2 cells were transfected with the plasmids overexpressing flag-Bdh1 or vector for 24 h, and then treated with vehicle or PA for 24 h. Bar: 50 μm. All results are representative of three independent experiments. Values are presented as mean ± SD. *P < 0.05; **P < 0.01; ***P < 0.001.

As the Bdh1 deficiency promoted inflammation and apoptosis by increase of ROS, we next sought to determine whether Bdh1 supplementation could protect LO2 cells from lipotoxicity. To this end, we transfected LO2 cells with flag-Bdh1 overexpression plasmid to block the PA-induced Bdh1 reduction (Fig. 3A). Notably, ROS assay showed that the Bdh1 overexpression nearly completely reversed the PA-induced ROS overproduction (Fig. 3B). JC-1 staining showed that the total ROS was mainly from mitochondrial ROS (Fig. 3C). As to inflammation, Bdh1 overexpression also reversed the PA-induced activation of IL-1β and IL-18 (Fig. 3D), as well as the mRNA levels of IL-1β and IL-18 (Fig. 3E). Moreover, PA-induced elevation of cleaved caspase 3 was also reversed by Bdh1 overexpression (Fig. 3F) and TUNEL assay further confirmed that Bdh1 overexpression reversed PA-induced apoptosis (Fig. 3G). These evidences suggest that Bdh1 may play a protective role in pathogenesis of MAFLD and hyperlipidemia-induced Bdh1 reduction in liver contribute to hepatocellular lipotoxicity.

### Bdh1 promotes Nrf2 nuclear translocation and activates Nrf2-mediated antioxidant pathway

**Fig. 4:**
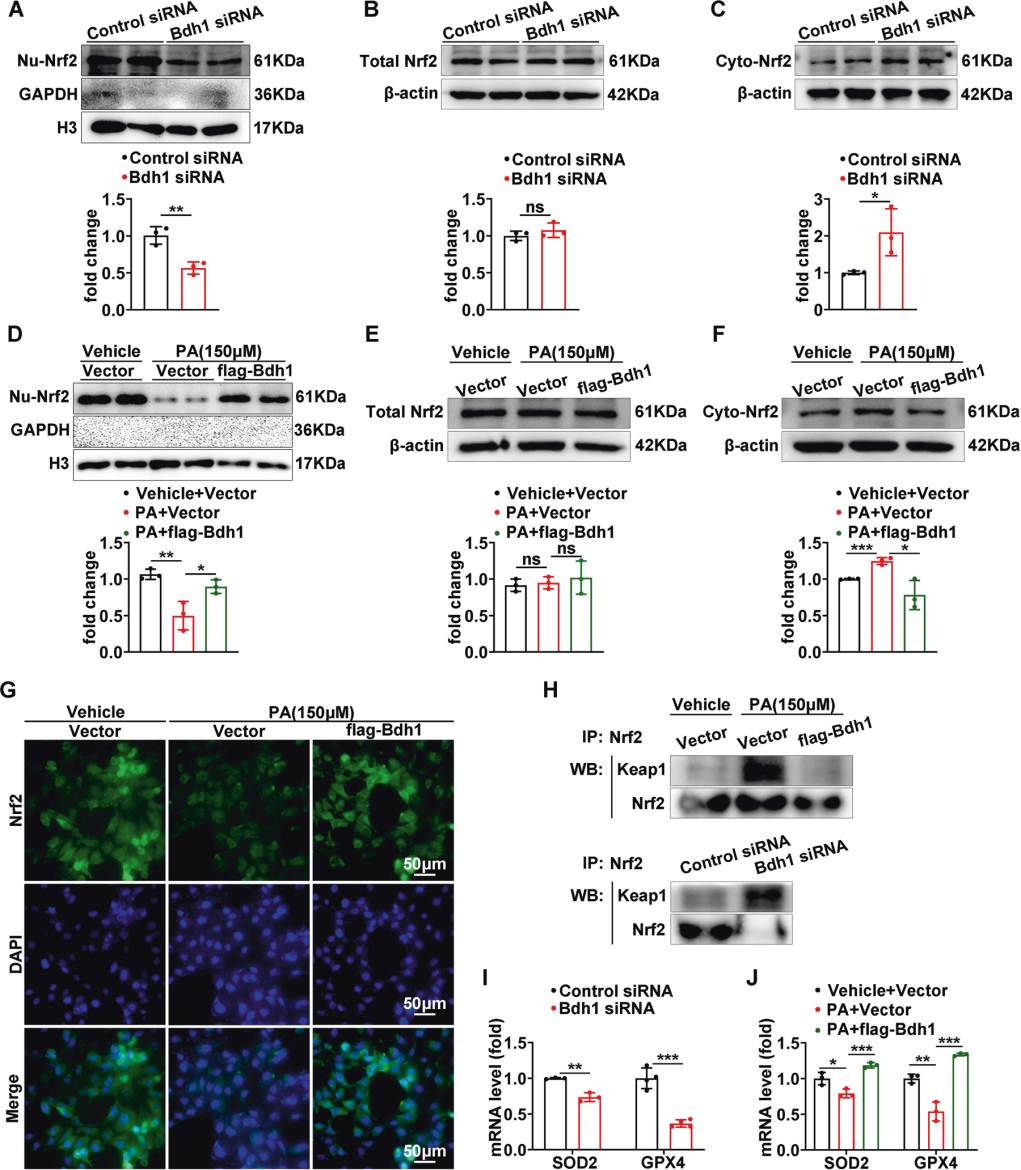
Bdh1 promotes Nrf2 nuclear translocation and Nrf2-mediated antioxidant pathway. **A**–**C** Representative western blots showing the protein level of Nrf2 protein in the nuclear extracts (**A**), cytosolic extracts (**B**), and total lysates (**C**) of LO2 cells transfected with control siRNA or Bdh1 siRNA. **D**–**F** Representative western blots showing the protein level of Nrf2 in the nuclear extracts (**D**), cytosolic extracts (**E**), and total lysates (**F**) of vetor and Bdh1-overexpressed LO2 cells treated by vehicle or PA. **G** Representative IF images showing the location of Nrf2 in vetor and Bdh1-overexpressed LO2 cells treated by PA. Bar: 50 μm. **H** Immunocoprecipitation showing the interaction between Keap1 and Nrf2 in LO2 cells with indicated treatment. **I** mRNA levels of GPX4 and SOD2 in LO2 cells transfected with control or Bdh1 siRNA. **J** mRNA levels of GPX4 and SOD2 in LO2 cells transfected with indicated treatment. LO2 cells were transfected with the plasmids overexpressing flag-Bdh1 or vector for 24 h, and then treated with vehicle or PA for 24 h. Bar: 50 μm. All results are representative of three independent experiments. Values are presented as mean ± SD. *P < 0.05; **P < 0.01; ***P < 0.001.

Given that Nrf2 is a well-known transcription factor that regulates transcriptional induction of ARE-containing genes encoding antioxidant enzymes in response to cellular stresses including ROS [17, 18], we next sought to determine whether Bdh1 mediates anti-ROS function through activation of Nrf2. As Nrf2 is a nuclear transcription factor, we detected the protein level of Nrf2 by western blot (WB) in nuclear extracts. Of note, in LO2 cells transfected with Bdh1 siRNA, the nuclear protein level of Nrf2 was significantly lower than that in cells transfected with control siRNA (Fig. 4A), whereas the Nrf2 protein level in total lysates was unchanged with an increase of cytosolic Nrf2 protein level in Bdh1 siRNA-transfected LO2 cells (Fig. 4B, C). Moreover, PA could induce Nrf2 reduction in nuclear, whereas Bdh1 overexpression could promote Nrf2 relocation in nuclear (Fig. 4D), which was further confirmed by Nrf2 nuclear translocation assay with immunostaining (Fig. 4G). In contrast, the Nrf2 protein level in total lysates was unchanged with a decrease of cytosolic Nrf2 protein level in Bdh1-overexpressed LO2 cells (Fig. 4E, F). Given that Keap1 is the most important regulator of Nrf2, we next determine whether Bdh1 regulates Nrf2 through Keap1. We performed immunoprecipitation and observed stronger interaction between Keap1 and Nrf2 in Bdh1 siRNA-transfected cells. On the contrary, Bdh1 overexpression reversed the PA-induced increase of binding between Keap1 and Nrf2 (Fig. 4H). In addition, the mRNA levels of GPX4 and SOD2, two of the classical downstream targets of Nrf2, were downregulated by Bdh1 knockdown (I), whereas they were upregulated by Bdh1 overexpression (J), which is consistent with the nuclear location of Nrf2.

### Bdh1 ameliorates PA-induced lipotoxicity through activation of Nrf2

**Fig. 5:**
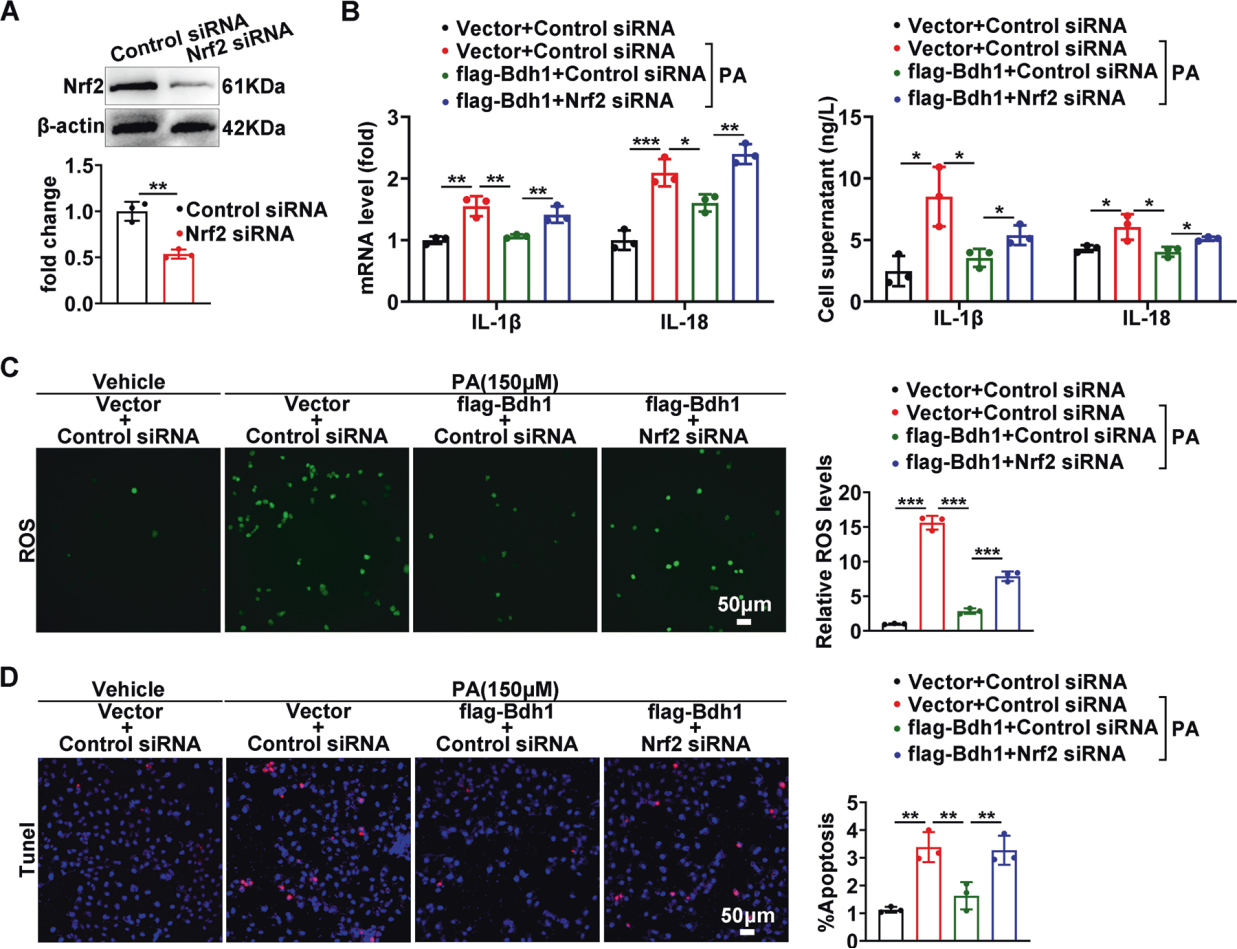
Bdh1 ameliorates PA-induced lipotoxicity through Nrf2. **A** Representative western blots showing the protein level of Nrf2 in LO2 cells transfected with control siRNA and Nrf2 siRNA. **B** mRNA levels (above) and cell culture supernatant concentrations (below) of IL-1β and IL-18 in LO2 cells with indicated treatment. **C** DCFH-DA probe was used to detect the level of ROS in LO2 cells with indicated treatment. **D** TUNEL assay showing the apoptosis level of LO2 cells with indicated treatment. Before indicated assay, LO2 cells were transfected with vector or flag-Bdh1 and control siRNA or Nrf2 siRNA for 24 h, and then treated with vehicle or PA for another 24 h. Bar: 50 μm. All results are representative of three independent experiments. Values are presented as mean ± SD. *P < 0.05; **P < 0.01; ***P < 0.001.

To determine whether Bdh1 ameliorates lipotoxicity by regulation of Nrf2, we next transfected Nrf2 siRNA into LO2 cells together with flag-Bdh1 plasmid under PA treatment (Fig. 5A). As shown in Fig. 5B, Nrf2 knockdown blocked the anti-inflammation effect of Bdh1 overexpression on PA-treated LO2 cells. Similarly, Nrf2 knockdown also blocked the anti-ROS effect of Bdh1 overexpression on PA-treated LO2 cells (Fig. 5C). Further TUNEL assay showed that LO2 cells transfected with flag-Bdh1 and Nrf2 siRNA exhibited much more apoptosis than that transfected with flag-Bdh1 and Control siRNA (Fig. 5D). These data indicate that Bdh1 mediated anti-lipotoxicity function through activation of Nrf2.

### Bdh1 activated Nrf2 by enhancing metabolic flux of fumarate production

**Fig. 6:**
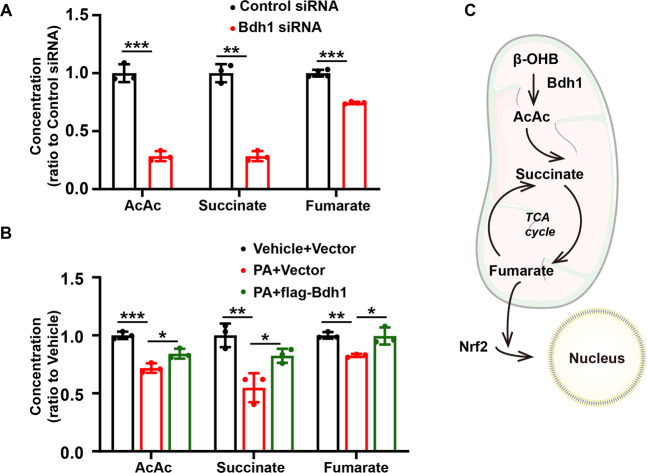
Bdh1-mediated βOHB metabolism promoted the fumarate production through AcAc-succinate-fumarate metabolic pathway. **A** The concentrations of AcAc, succinate, and fumarate in LO2 cells transfected with control siRNA or Bdh1 siRNA. **B** The concentrations of AcAc, succinate, and fumarate in vector or Bdh1-overexpressed LO2 cells stimulated by vehicle or PA (D). **C** Scheme showing the Bdh1-regulated metabolic flux composed of βOHB-AcAc-succinate-fumarate. All results are representative of three independent experiments. Values are presented as mean ± SD. *P < 0.05; **P < 0.01; ***P < 0.001.

In Bdh1-mediated βOHB metabolism pathway, Bdh1 firstly metabolites βOHB into AcAc, which could enter into TCA cycle and then is metabolized into Succinate and fumarate in turn. As the fumarate is a well-known activator of Nrf2 signaling, we next investigated whether Bdh1 activated Nrf2 by increase of fumarate. As expected, we found that the concentrations of AcAc, succinate, and fumarate were all decreased in LO2 cells transfected with Bdh1 siRNA (Fig. 6A). Similarly, in Bdh1 siRNA-transfected LO2 cells, PA treatment could also reduce the levels of AcAc, Succinate and fumarate, which was successfully blocked by Bdh1 overexpression (Fig. 6B). These findings collectively reveal a metabolic flux composed of βOHB-AcAc-succinate-fumarate, which could be regulated by Bdh1 and affected the downstream Nrf2 signaling (Fig. 6C).

### AAV-mediated Bdh1 hepatic expression alleviated the progression of MAFLD

**Fig. 7:**
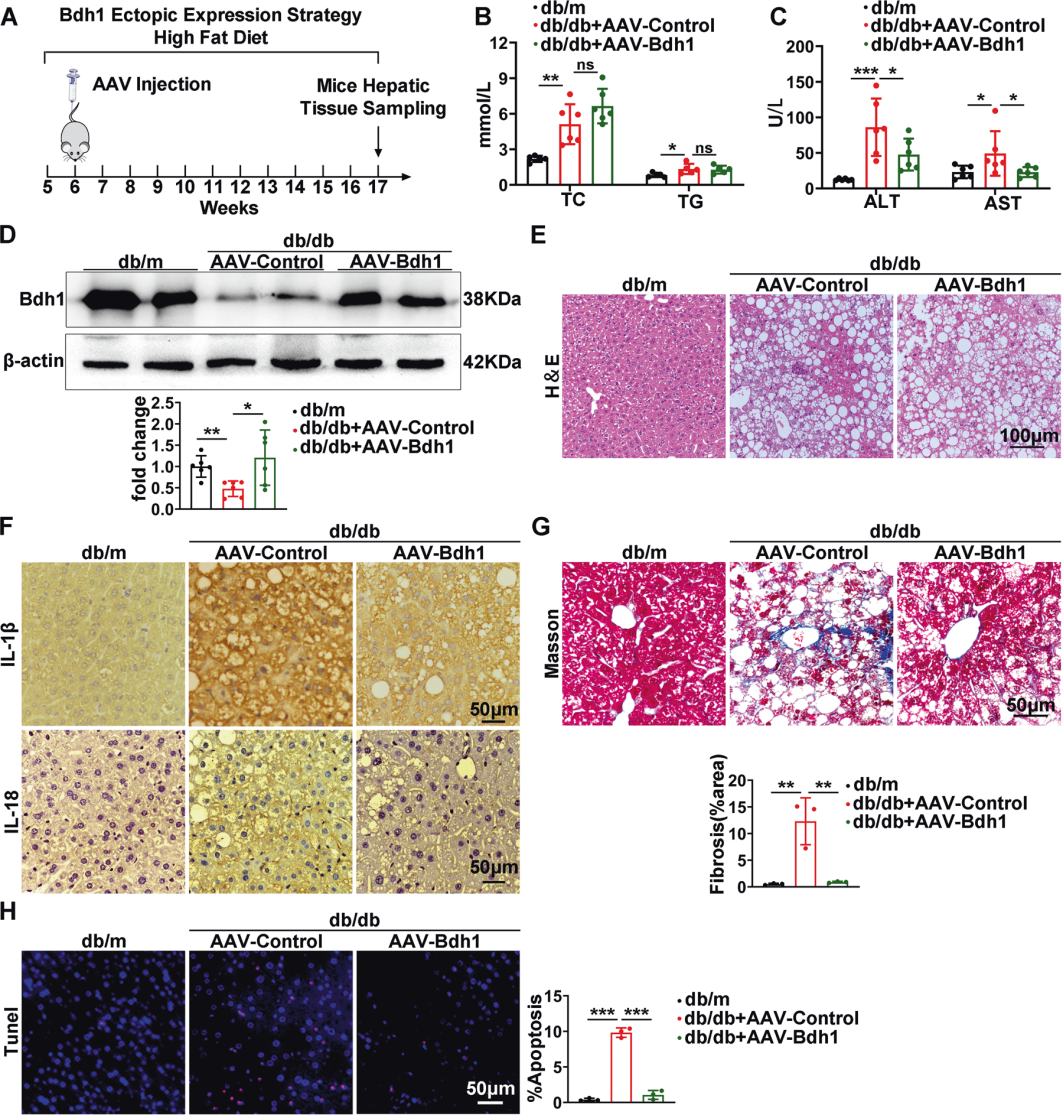
AAV-mediated Bdh1 hepatic expression alleviated the progression of MAFLD. **A** A schematic representation of the Bdh1 ectopic expression strategy in db/db mice. **B** Serum levels of TG and TC in db/m and db/db mice with vector or AAV-Bdh1 injection (*n* = 6 per group). **C** Serum levels of ALT and AST in db/m and db/db mice with vector or AAV-Bdh1 injection (*n* = 6 per group). **D** Representative western blots showing the protein level of Bdh1 in livers from indicated groups (*n* = 5 per group). (**F**–**H**) The representative photomicrographs of H&E (**E**), IHC (IL-1β, IL-18) (**F**), Masson (**G**), and Tunel staining (**H**) show the pathological changes in livers from indicated groups. Bar: 100 μm in E, 50 μm in **F**–**H**. All results are representative of three independent experiments. Values are presented as mean ± SD. *P < 0.05; **P < 0.01; ***P < 0.001.

On the basis of the pronounced capacity of Bdh1 to inhibit ROS overproduction and inflammation, we next explored the therapeutic efficacy of Bdh1 expression in db/db mice, a MAFLD mouse model. The experimental strategy is shown in Fig. 7A. At the time point of 12 weeks after injection of the control or Bdh1-encoding virus, we performed serological tests for TC, TG, AST, and ALT, which are the most important function indicator of liver. Notably, we found that there was no significant difference in serum level of TC and TG between AAV-Bdh1-injected and AAV-Control-injected db/db mice (Fig. 7B), whereas the serum levels of AST and ALT in AAV-Bdh1-injected db/db mice were lower than that in control mice (Fig. 7C), indicating that AAV-Bdh1 injection ameliorates the liver injury in MAFLD. To confirm whether mouse Bdh1 was effectively expressed in the livers with AAV-Bdh1 injection, we performed western blot assay. As expected, we observed increased Bdh1 expression in livers from AAV-Bdh1 injected mice than that in AAV-control injected mice (Fig. 7D). In further histological analysis, H&E staining showed that hepatic morphology of livers from AAV-Bdh1 injected db/db mice was similar with that in AAV-control injected mice, which is consistent with the unchanged TC and TG (Fig. 7E). Notably, Masson staining showed AAV-Bdh1 injection significantly reduced the MAFLD-related liver fibrosis. In addition, the inflammation and apoptosis were also substantially reduced by AAV-Bdh1 injection (Fig. 7F–H). These findings collectively provide strong support for the promising application of Bdh1 as a therapeutic target in MAFLD.

## Discussion

To date, as no effective drugs are clinically available, the only therapeutic choices for MAFLD are lifestyle interventions and weight loss. In this study, we identified Bdh1 as a novel therapeutic target for MAFLD. Our data showed that expression of Bdh1 was reduced in the fatty liver of MAFLD mouse model and PA-treated LO2 cells. In vitro, Bdh1 overexpression could protect LO2 cells from lipotoxicity. And in vivo, AAV-mediated Bdh1 hepatic expression reversed the fibrosis, inflammation, and apoptosis in MAFLD. Mechanistically, Bdh1-mediated βOHB metabolism inhibits oxidative stress by activation of Nrf2 through upregulation of fumarate production. Thus, Bdh1-mediated βOHB metabolism in liver lights a new way for MAFLD treatment.

Because oxidative stress is considered as the dominant contributor to the disease progression in MAFLD [11], the efficacy of antioxidants such as vitamin E has been assessed in clinical trials for treatment of fatty liver disease [27, 28]. Although the patients with vitamin E treatment exhibited a decrease in MAFLD activity score with improved liver enzymes, steatosis, and inflammation [29], vitamin E is not recommended to diabetic NASH patients because of adverse effects, including increased risk of all-cause mortality, prostate cancer, and hemorrhagic stroke [30]. Besides, another ROS antioxidant Nrf2 is also targeted to inhibit oxidative stress by relevant activators in MAFLD mouse model [31, 32], whereas no approved pharmacological therapies are developed for clinical use [33]. Thus, more specific mediators of MAFLD are needed to be demonstrated for precise targeting. In this study, we identified Bdh1 as another therapeutic target in progression of MAFLD. We showed that Bdh1 overexpression activated Nrf2 by increasing fumarate production through metabolic flux composed of βOHB-AcAc-succinate-fumarate (Fig. 6). In contrast to compound inhibitors which directly bind targets to block the function, Bdh1 indirectly regulates the Nrf2-mediated antioxidant pathway with a series of biological processes. We suppose that reversing the disease-induced imbalance of gene expression, for instance, the hyperlipidemia-induced Bdh1 downregulation, is more reasonable than hastily inhibiting one or two members of oxidative stress pathway in the treatment of MAFLD.

As a metabolic fuel for the tricarboxylic acid (TCA) cycle, ketone bodies will be released into blood from liver and transported to extrahepatic organs such as the brain, heart, and kidneys in the state of prolonged fasting [21]. Ketogenesis diet (KD), which could increase endogenous ketone bodies, was first introduced by doctors as a therapeutic method for many diseases [34, 35]. However, unlike the beneficial effect of KD on extrahepatic tissues in T2DM [36–39], KD feeding promotes hepatic lipid accumulation and leads to liver damage in a T2DM mouse model [40]. Thus, the role of ketone body in liver might be very different from extrahepatic organs. As the rate-limiting enzyme of ketone body metabolism, the functions of Bdh1 in fatty liver is unknown. In this study, we observed Bdh1 reduction in liver from a T2DM mouse model and PA-treated LO2 cells. Notably, overexpression of Bdh1 in PA-treated LO2 cells or fatty livers could protect hepatocytes from lipotoxicity (Fig. 3), which is consistent with the protective effect of Bdh1 in TAC-induced heart failure [24]. Interestingly, Bdh1 knockout led to lipid accumulation in liver during fasting [25], whereas in our study, Bdh1 hepatic overexpression didn’t affect lipid metaboism in liver of MAFLD mouse model (Fig. 7). In a word, the role of KD might very differs from that of Bdh1 in liver and Bdh1 would functions as different roles in different physiological conditions. Given that Bdh1 can also mediates β-hydroxybutyrylation to regulate gene expression in hepatocellular carcinoma stem cells [26], continued studies aimed at identifying new mechanisms mediated by Bdh1 in MAFLD are desperately needed.

**Fig. 8:**
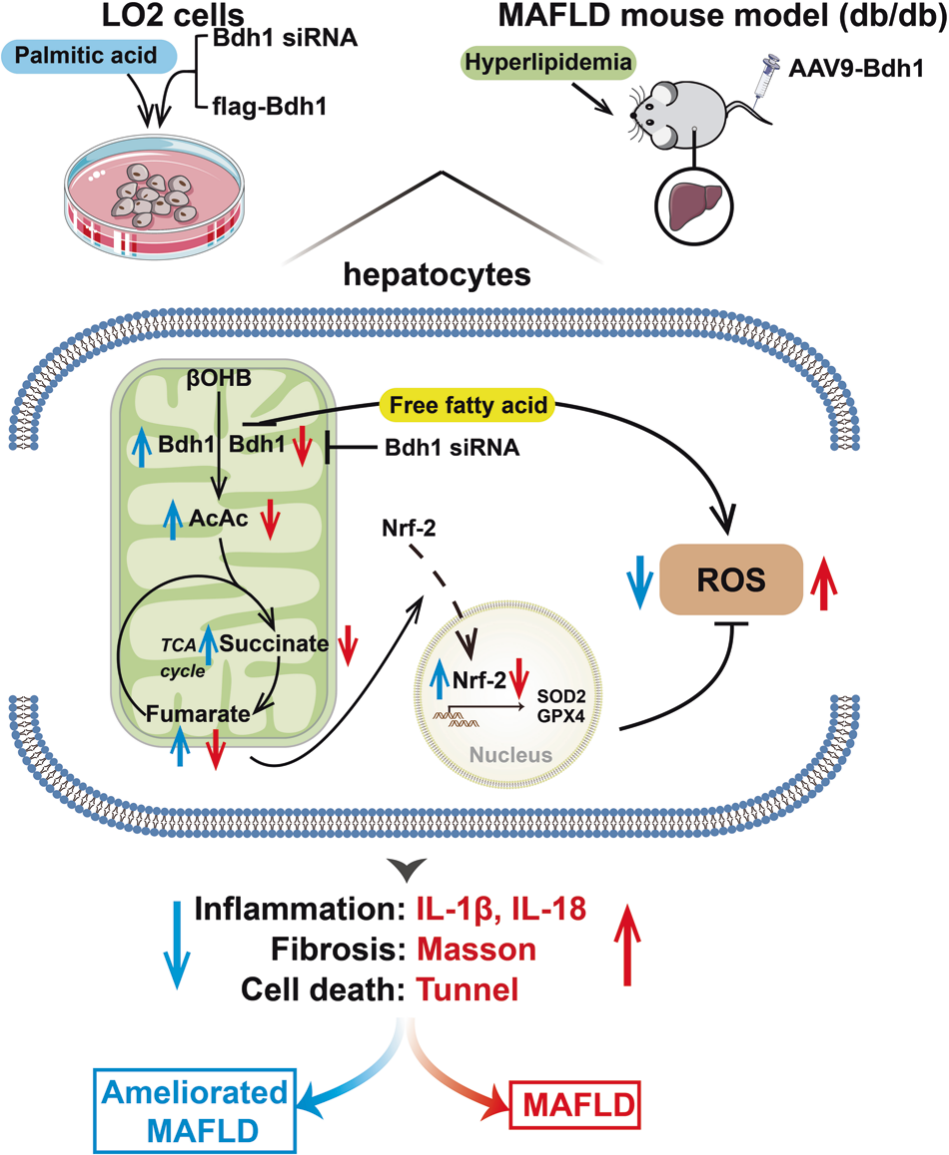
Schematic diagram depicting the mechanism by which Bdh1-mediated βOHB metabolism ameliorates MAFLD. Hyperlipidemiain db/db mice or palmitic acid treatment in LO2 cells leads to reduction of Bdh1 expression, which subsequently downregulates the fumarate level by metabolic flux composed of βOHB-AcAc-Succinate-Fumarate. The reduced fumarate level decreases nuclear translocalization of Nrf2, inducing overproduction of ROS, which finally activates the inflammation, fibrosis and apoptosis in MAFLD. In addition, overexpression of Bdh1 in LO2 cells or db/db mice could reverse the pathological injury by increasing fumarate/Nrf2/ROS axis.

In conclusion, our results provide evidence for the proposed mechanism depicted in Fig. 8. Hyperlipidemia in db/db mice leads to downregulated expression of Bdh1, which subsequently downregulates the fumarate level by metabolic flux composed of βOHB-AcAc-succinate-fumarate. The reduced fumarate level decreases nuclear translocalization of Nrf2, inducing overproduction of ROS, which finally activates the inflammation, fibrosis, and apoptosis in fatty liver. Our findings also highlight the possibility of Bdh1 hepatic expression in attenuating MAFLD.

## Materials and methods

### Animals

Five-week-old male db/m and db/db mice were purchased from GemPharmatech Co., Ltd. (Nanjing, China). 60% high-fat diet (HFD) and KD (74.2% fat, 8.9% protein, 3.2% carbohydrate) were purchased from Trophic Animal Feed High-tech Co., Ltd. (Jiangsu, China). All animal experiments were performed under the following condition: room temperature 23 ± 1°C, relative humidity 60% ± 10%, and an alternating 12 h light–dark cycle in individually ventilated cages. Animal experiments were approved by the Institutional Animals Ethics Committees of Southwest Medical University and in accordance with the National Institutes of Health (NIH) guidelines for the care and use of laboratory animals.

### Human liver samples

Human liver samples were obtained from patients with or without MAFLD who had undergone either liver biopsy or liver resection because of a liver cancer from pathology department. All procedures that involved human samples were approved by the Affiliated Hospital of Southwest Medical University Ethics Committee and were performed in a manner consistent with the principles outlined in the Declaration of Helsinki. Informed consent was obtained from all subjects.

### Animal experiments

For AAV9-mediated Bdh1 hepatic expression, 100 μL AAV9-Bdh1 (3.40E + 12vg/mL, Beijing Syngentech Co., LTD. China) or the negative control (1.90E + 13vg/mL) were injected into db/db mice *via* the caudal vein. Body weight and blood glucose were recorded weekly.

### LO2 Cell culture

LO2 cells (ATCC, USA) were cultured in high glucose Dulbecco’s Modified Eagle Medium (HyClone, Logan, Utah, USA) containing 10% fetal bovine serum (Sciencell, USA) and supplemented with 1% penicillin–streptomycin (Beyotime, Shanghai, China). LO2 cells were cultured at 37°C with 5% CO2 until 60–70% confluence, cells were exposed to normal control (vehicle) and palmitic acid (Sigma-Aldrich, Saint Louis, MO, USA) for 48 h. PA was prepared as previously described [41].

### Histological assay

The liver tissues were fixed in 4% paraformaldehyde for 24 h, embedded in paraffin, and sectioned at 4 μm thickness. The sections were stained by the hematoxylin–eosin (H&E), or Masson-trichrome methods for light microscopic analysis and morphometry.

### Immunohistochemistry staining

Briefly, 4-μm-thick paraffin sections were dewaxing hydration and stained with primary antibodies against IL-18 (Abcam, UK), IL-1β (Cell Signaling Technology, USA). The sections were stained with biotin-labeled goat anti-rabbit IgG or biotin-labeled anti-mouse IgG and then treated with the Horseradish enzyme-labeled ovalbumin of Streptomyces (Beijing ZSGB Biological Technology CO., LTD. China). Each photograph of the stained sections was scanned using a light microscope.

### ROS, JC-1and TUNEL assay

The level of ROS in LO2 cells was measured by DCFH-DA fluorescent probe according to the ROS Assay Kit protocol (Beyotime, China). TUNEL staining for the liver paraffin sections was performed according to the TUNEL Kit protocol (Roche, USA). JC-1 staining for LO2 cells was performed according to the JC-1 assay Kit protocol (Solarbio, China).

### Human Bdh1 cDNA transfection

The human Bdh1-overexpressed plasmid (pCMV3-Bdh1-Flag) and the vector plasmid (pCMV3) were purchased from Sino Biological Inc. (Beijing, China) and transfected into LO2 cells with Lipofectamine 3000 (Invitrogen).

### siRNA transfection

The siRNAs for Bdh1 and Nrf2 were purchased from RiboBio (Guangzhou, China). The Bdh1 and control siRNA were transfected into LO2 cells with ribo*FECT*^TM^ CP Reagent and ribo*FECT*^TM^ CP Buffer (RiboBio, Guangzhou, China).

### Measurement of AcAc, succinate, and fumarate

The AcAc content in LO2 cells was measured by human acetoacetate ELISA Kit (Shanghai J&L Biological, China). The succinate and fumarate content of LO2 cells were measured using the Succinate Assay Kit (Sigma-Aldrich, Saint Louis, MO, USA) and the Fumarate Assay Kit (Sigma-Aldrich, Saint Louis, MO, USA) according to the manufacturer’s instructions.

### qRT-PCR analysis

Total RNAs of liver tissue and LO2 cells were extracted with Trizol (Invitrogen, USA). The ReverTra Ace qPCR RT Master Mix (FSQ-201, TOYOBO) was used for reverse transcription reaction and QuantiNova SYBR Green PCR Kit (QIAGEN, German) was used for qRT-PCR. The qRT-PCR was performed with Analytikjena qTOWER 3 G real-time PCR system (JENA, German) according to the manufacturer’s instructions. Primers used in this study were shown as below: hBdh1:5′GACAGCCTAAACAGTGACCGA3′;5′GAGCGGACAATCTCCACCA3′, mBdh1:5′CGGCTAGTGGCAAAGCTATC3′;5′GTTGC- AGACATTGAGCTGGA3′, hIL-1β:5′TTCGACACATGGGATAACGA- GG3′;5′TTTTTGCTGTGAGTCCCGGAG3′,hIL-18:5′TCTTCATTGAC-CAAGGAAATCGG3′;5′TCCGGGGTGCATTATCTCTAC3′mGAPDH:5′AGGTCGGTGTGAACGGATTTG3′;5′TGTAGACCATGTAGTTGAGGTCA3′,hGAPDH:5′CAATGACCCCTTCATTGACC3′;5′GACAAGC-TTCCCGTTCTCAG3′,hSOD2:5′GCTCCGGTTTTGGGGTATCTG3′;5′GCGTTGATGTGAGGTTCCAG3′,hGPX4:5′GAGGCAAGACCGAAGTAAACTAC3′;5′CCGAACTGGTTACACGGGAA3′. GAPDH was used as an internal reference gene to normalize target gene expression. All the samples were used in triplicates. The 2^−△△Ct^ method was used to calculate the relative gene expression in comparison with the reference gene.

### Immunoprecipitation and western blot analysis

For immunoprecipitation (IP), cells were suspended in lysis buffer (50 mmol/L Tris-Cl (pH 7.4), 150 mmol/L NaCl, 5% glycerol, 1% NP-40, 1 mmol/L EDTA), supplemented with 1 mM PMSF and 4 μg/mL protease inhibitor cocktail (Sigma). Lysates were centrifuged at 13,000 g for 10 min, and the supernatant was added to 2 μL indicated antibodies and 100 μL protein A agarose beads (Invitrogen) to incubate for 4 h at 4 °C. Afterward, protein A beads were washed with 250 mmol NaCl four times. For western blot, total proteins of liver tissue and LO2 cells were extracted with extraction buffer (RIPA). Nuclear proteins were extracted with the Nucleoprotein Extraction Kit protocol (Shanghai Sangon Biotech, China). The protein samples were separated by sodium dodecyl sulfate-polyacrylamide gel electrophoresis (SDS-PAGE) and transferred into a PVDF membrane (Millipore). The membranes were incubated with 5% BSA to block other contaminants, and then with primary antibodies. Immunoblotting was performed using anti-Bdh1 antibody (Abcam, UK), anti-Nrf2 antibody and anti-Histone H3 antibody (CST, USA), anti-cleaved caspase 3 antibody, anti-IL-18 antibody, anti-IL-1β antibody, anti-GAPDH antibody, and anti-β actin antibody (Beyotime, China).

### Serum measurements

Serum TC, TG, ALT, and AST levels were determined in mouse serum using TC, TG, ALT, and AST Detection Kits (Nanjing Jiancheng Bio, Nanjing, China) by an automatic biochemical analyzer.

### Statistical analysis

Data are expressed as the means ± standard deviation (SD). The student’s *t*-test was employed for comparisons between two groups. Differences were evaluated using GraphPad Prism9. *P* < 0.05 was considered statistically significant. The statistical significance was *P < 0.05, **0.001 < *P* < 0.01; ***P < 0.001.

## Data Availability

All data generated or analyzed during this study are included in this published article.
